# Regulation of mitochondrial dynamics: convergences and divergences between yeast and vertebrates

**DOI:** 10.1007/s00018-012-1066-6

**Published:** 2012-07-18

**Authors:** Jian Zhao, Urban Lendahl, Monica Nistér

**Affiliations:** 1 Department of Oncology-Pathology, Karolinska Institutet, CCK R8:05, Karolinska University Hospital Solna, 171 76 Stockholm, Sweden; 2 Department of Cell and Molecular Biology, Karolinska Institutet, 171 77 Stockholm, Sweden

**Keywords:** Mitochondria, Mitochondrial dynamics, Mitochondrial fusion/fission, Yeast, Vertebrates

## Abstract

In eukaryotic cells, the shape of mitochondria can be tuned to various physiological conditions by a balance of fusion and fission processes termed mitochondrial dynamics. Mitochondrial dynamics controls not only the morphology but also the function of mitochondria, and therefore is crucial in many aspects of a cell’s life. Consequently, dysfunction of mitochondrial dynamics has been implicated in a variety of human diseases including cancer. Several proteins important for mitochondrial fusion and fission have been discovered over the past decade. However, there is emerging evidence that there are as yet unidentified proteins important for these processes and that the fusion/fission machinery is not completely conserved between yeast and vertebrates. The recent characterization of several mammalian proteins important for the process that were not conserved in yeast, may indicate that the molecular mechanisms regulating and controlling the morphology and function of mitochondria are more elaborate and complex in vertebrates. This difference could possibly be a consequence of different needs in the different cell types of multicellular organisms. Here, we review recent advances in the field of mitochondrial dynamics. We highlight and discuss the mechanisms regulating recruitment of cytosolic Drp1 to the mitochondrial outer membrane by Fis1, Mff, and MIEF1 in mammals and the divergences in regulation of mitochondrial dynamics between yeast and vertebrates.

## Introduction

**Fig. 1:**
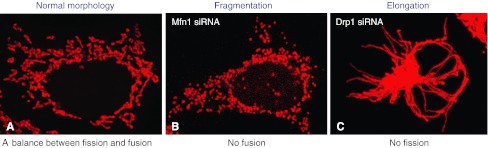
Mitochondrial morphology is regulated by a balance between fission and fusion. **a** The normal morphology of mitochondria is a mixed reticulum with tubular and round forms as shown in 293T cells. **b** The absence of fusion by depletion of Mfn1 using siRNA leads to mitochondrial fragmentation. **c** The absence of fission by depletion of Drp1 using siRNA leads to mitochondrial elongation

Mitochondria are double membrane-bound organelles that play a crucial role in energy metabolism producing ATP through oxidative phosphorylation. Besides this essential function, these organelles are also involved in many important cellular processes such as β-oxidation of fatty acids, the urea cycle, and the generation and detoxification of reactive oxygen species (ROS). Mitochondria are crucial for the regulation of cell proliferation, differentiation, and intracellular calcium homeostasis, and they are also key players in the regulation of cell death pathways. Mitochondria are highly dynamic organelles that, upon the cell’s metabolic demands or pathological conditions, frequently change their shape. A number of mitochondria-shaping proteins control mitochondrial fission and fusion events, leading to a continuous remodeling of mitochondrial networks: increased fission or decreased fusion can lead to mitochondrial fragmentation, whereas increased fusion or decreased fission can lead to mitochondrial elongation (Fig. 1) [1–3]. Mitochondrial dynamics involves the shape, size, distribution, transport and number of mitochondria in the cell and is controlled by a balance between mitochondrial fusion and fission events [4, 5]. Even in unicellular organisms, such as in yeast, mitochondria show a highly complicated and dynamic behavior. An increasing number of studies suggest that mitochondrial dynamics plays a critical role in controlling mitochondrial function, and it has been suggested that mitochondrial morphology reflects their functional status. Therefore, imbalance of mitochondrial fission and fusion events impacts on a broad range of cellular biological processes. The carefully orchestrated balance between mitochondrial fission and fusion is also crucial for maintaining a healthy population of mitochondria. Mitochondrial fusion allows mitochondria to exchange their content including the mitochondrial DNA (mtDNA) and proteins between individual mitochondria, whereas mitochondrial fission allows for mitochondrial biogenesis, and the segregation of damaged and inactive mitochondria by autophagic clearance [6]. Abnormal mitochondrial dynamics is closely associated with mitochondrial dysfunction, involving the regulation of bioenergy metabolism, Ca2^+^ signaling, ROS production, maintenance of mtDNA, mitochondrial biogenesis and transport, in turn impacting on a wide range of cellular processes, including programmed cell death, apoptosis resistance of cells, autophagy, cell-cycle regulation, nuclear DNA integrity, cell proliferation, differentiation and senescence, but the specific molecular mechanisms involved are far from clear [4, 7–12]. Several key mitochondria-shaping proteins have been demonstrated to be essential for mice embryonic and brain development; for example, mice lacking Mfn1 (mitofusin 1), Mfn2 (mitofusin 2), OPA1 (optic atrophy 1) or Drp1 (dynamin-related protein 1) die at an early embryonic stage [13–16]. Similarly, a lethal mutation of the Drp1 gene was reported in a neonate with abnormal brain development and optic atrophy [17]. These data further emphasize the crucial role of mitochondria-shaping proteins in the development of mammals. Dysfunction of mitochondrial dynamics has been implicated in embryonic developmental processes [13–15], aging [8, 18] and a wide range of pathological conditions [12, 19], including neurodegenerative diseases [4, 6, 20], diabetes [21, 22], cardiovascular disease [23], muscle atrophy [24, 25] and cancer [26].

The budding yeast *Saccharomyces cerevisiae* is a favorite model system to study mitochondrial dynamics due to its many experimental advantages [3]. Genetic screens in yeast have identified a set of proteins required for the maintenance of mitochondrial morphology. Some of the components mediating mitochondrial fusion and fission are conserved from yeast to human (see Tables 1 and 2), indicating that the fundamental mechanisms controlling mitochondrial dynamics have been maintained during evolution. It should be stressed, however, that the mitochondrion is more complex in humans than in yeast, and that there are many more proteins in human mitochondria [27]. Proteomic analyses of both the budding yeast *S. cerevisiae* and human-heart mitochondria indicate that these mitochondria contain ~1,000 and ~1,500 different proteins in yeast and human, respectively. Nuclear DNA encodes most of these proteins (about 99 %), whereas only a minor fraction (eight proteins in budding yeast and 13 in humans) are encoded by mitochondrial DNA (mtDNA) [27, 28]. Depending on the complexity of differentiated mammalian cells in different tissues and their metabolic state, human mitochondria contain about 1,000–2,000 distinct proteins in different cell types [28]. Although many mitochondrial metabolic pathways in yeast and human share a set of conserved proteins, it is believed that there are major differences in the regulation of mitochondrial functions between yeast and human. During the last two decades, considerable progress has been made in our understanding of mitochondrial dynamics due to its importance for many normal biological processes in cells and its involvement in human diseases [2, 3, 12, 29]. Although most core components of the mitochondrial fusion and fission machineries are evolutionarily conserved (see Tables 1 and 2), a growing number of novel mammalian proteins that regulate mitochondrial dynamics have been identified, suggesting that the regulatory mechanisms for mitochondrial dynamics have become evolutionarily more sophisticated in mammals than in unicellular organisms such as yeast. However, we are only beginning to understand the diversity of mitochondrial dynamics between yeast and mammals.

**Table 1:** Proteins involved in mitochondrial fission in yeast and mammals

Proteins	Yeast	Mammals	Subcellular localization	Known or possible functions in mitochondrial dynamics	References
Key players	Dnm1p	Drp1	Cytosol and MOM-associated	Dynamin-related GTPase for fission of the outer mitochondrial membrane	[31], [53]
	Fis1p	Fis1	MOM-anchored	Receptor for recruitment of Dnm1p/Drp1 to mitochondria, promoting fission	[41], [56]
	Mdv1p	–	Cytosol and MOM-associated	Adaptor binding to Fis1p for recruitment of Dnm1p to mitochondria	[40]
	Caf4p	–	Cytosol and MOM-associated	Adaptor binding to Fis1p for recruitment of Dnm1p to mitochondria	[34]
	–	Mff	MOM-anchored	Receptor for recruiting Drp1 to mitochondria, promoting fission	[65]
	-	MIEF1(MiD51)/ MiD49	MOM-anchored	Receptor for recruiting Drp1 to mitochondria, inhibiting Drp1 function	[68], [69]
Regulators	Num1p	–	Cell cortex and MOM-associated	A role in the recruitment or stability of Dnm1p on mitochondria	[49]
	Mdm36p	–	MOM-associated	Recruiting Dnm1p to mitochondria	[50], [48]
	Mdm33p	–	MIM-anchored	Inner membrane fission	[51]
	–	Endophilin B1	Cytosol and MOM-associated	Outer membrane fission	[70]
	–	GDAP1	MOM-anchored	Outer membrane fission	[73]
	–	MTP18	MIM-associated	Inner membrane fission	[72]
	MGR2?	MTGM	MIM-anchored	Inner membrane fission	[74]
Post-translational modifications	–	MARCH-V/MITOL /MARCH5	MOM-anchored	E3 ubiquitin ligase for Drp1 and hFis1	[86], [85], [87]
	–	Parkin	Cytosol and MOM-associated	E3 ubiquitin ligase for Drp1, regulating mitochondrial dynamics	[153]
	–	PINK1	Cytosol and MOM-anchored	A mitochondrial kinase, recruiting Parkin to mitochondria	[146], [155]
	–	Cyclin B/CDK1	Cytosol and MOM-associated	Mitotic phosphorylation of Drp1	[77]
	–	CaMKIα	Cytosol	Ca2^+^-dependent phosphorylation of Drp1	[80]
	–	Calcineurin (PP2B)	Cytosol and MOM-associated	Ca2^+^-dependent dephosphorylation of Drp1	[78], [81]
	–	PKA	Cytosol and MOM-associated	cAMP-dependent phosphorylation of Drp1	[190], [79]
	–	SENP5	Nucleus and Cytosol	SUMO protease for deSUMOylation of Drp1	[84]
	–	MAPL	MOM-anchored	SUMO ligase for SUMOylation of Drp1	[83]
	–	SUMO1	Nucleus, Cytosol and MOM-associated	SUMOylation of Drp1	[82]
	–	UBC9	Cytosol	SUMO-conjugating enzyme 9 for SUMOylation of Drp1	[82]

*–* No potential homologue has been identified in either yeast or mammals; *?* The roles in regulating mitochondrial dynamics are currently unclear. *MOM* Mitochondrial outer membrane, *MIM* mitochondrial inner membrane

**Table 2:** Proteins involved in mitochondrial fusion in yeast and mammals

Proteins	Yeast	Mammals	Subcellular localization	Known or possible functions in mitochondrial dynamics	References
Key players	Fzo1p	Mfn1/2	MOM-anchored	Dynamin-related GTPase for tethering and fusion of outer mitochondrial membrane	[93], [13]
	Mgm1p	OPA1	MIM-anchored, IMS	Dynamin-related GTPase required for fusion of the inner mitochondrial membrane	[102], [122]
	Ugo1p	–	MOM-anchored	Interaction with Fzo1p and Mgm1p to link the inner and outer membrane for fusion	[107]
	–	MIEF1	MOM-anchored	Promoting mitochondrial fusion in a Mfn2-independent manner	[68]
Regulators	–	MICS1/GHITM	MIM-anchored	Required for the mitochondrial tubular network and cristae organization	[143]
	–	MIB/VAT1	Cytosol and MOM-associated	Interacting with Mfn1/2 and negatively regulating Mfn1-dependent fusion	[139]
	–	Stoml2/SLP2	IMS/MIM-associated	Mfn2-binding protein required for stress-induced mitochondrial hyperfusion	[141], [140]
	–	BAX and BAK	Cytoplasm and MOM-associated	Activating assembly of Mfn2 complexes	[191]
	–	mitoPLD	MOM-anchored	Regulating mitochondrial fusion	[142]
	Mdm38p	LETM1	MIM-anchored	Required for the mitochondrial tubular network and cristae organization	[144], [145]
Post-translational modifications	Pcp1p/Rbd1p	PARL	IMS, MIM-anchored	Processing of Mgm1p/OPA1	[104], [105]
	Ups1p	PRELI	IMS, MIM-associated	Processing of Mgm1p/OPA1	[192], [115]
	Yme1p?	Yme1L	IMS	Processing of OPA1	[135], [133], [132]
	Oma1p?	OMA1/MPRP1	MIM-anchored	Metalloprotease, OMA1 involved in processing of OPA1	[134], [129]
	Phb2p?	PHB2	MIM-anchored or MIM-associated	Required for controlling the stability and proper processing of OPA1	[138], [136]
	–	Paraplegin	MIM-anchored	Processing of OPA1	[131]
	–	AFG3L1	MIM-anchored?	Processing of OPA1	[129]
	–	AFG3L2	MIM-anchored?	Processing of OPA1	[129]
	Mdm30p	–	Cytosol and MOM-associated	Ubiquitin ligase for ubiquitination of Fzo1p	[112]
	–	MARCH-V/MITOL /MARCH5	MOM-anchored	Ubiquitin ligase for ubiquitination of Mfn1	[88]
	–	Parkin	Cytosol and MOM-associated	Ubiquitin ligase for ubiquitination of Mfn1/2 upon induction of mitophagy	[163], [164]
	Ubp16p?	USP30	MOM-anchored	Deubiquitinating enzyme, USP30 involved in maintaining mitochondrial morphology	[193], [194]

– No potential homologue has been identified in either yeast or mammals; *?* Possible roles in regulating mitochondrial dynamics are currently unclear, *MOM* mitochondrial outer membrane, *MIM* mitochondrial inner membrane, *IMS* intermembrane space

There have been a number of excellent reviews published in the field of mitochondrial dynamics [1–3, 10, 12, 29, 30]. In this review, we highlight new advances in our understanding of mitochondrial dynamics processes in mammalian cells. Key players and regulators involved in mitochondrial fusion and fission pathways will be described in both yeast and vertebrates. Convergences and divergences of mitochondrial dynamics processes between yeast and vertebrates and molecular models for the regulation of these processes are discussed. In particular, several recently identified vertebrate-specific mitochondria-shaping proteins are introduced. We also highlight the possible roles of dysfunctional mitochondrial dynamics in human cancer.

## Mitochondrial fission in yeast and vertebrates

### The key players in the yeast mitochondrial fission machinery

The molecular mechanisms involved in the mitochondrial fission machinery are best understood in the budding yeast *S. cerevisiae*. The key fission-promoting protein Dnm1p, together with Fis1p (fission 1 protein) and Mdv1p (mitochondrial division 1 protein) are known to be essential components of the yeast fission machinery [2, 3, 29, 30].

#### Dnm1p

The role of Dnm1p in mitochondrial fission was first discovered in a screen for yeast mutants with defective mitochondrial morphology [31]. Dnm1p is a dynamin-related GTPase and a key component of the mitochondrial fission machinery in yeast. Dnm1p molecules are assembled in punctate structures that are primarily associated with the surface of the mitochondrial outer membrane [31–33], but some of the punctate structures are also found in the cytoplasm [31, 34, 35]. Recruitment of Dnm1p from the cytoplasm to mitochondria and assembly of Dnm1p along the mitochondrial surface at constriction sites is believed to be crucial for mitochondrial fission in yeast cells. However, most of the assembly complexes of Dnm1p along mitochondrial tubules are abortive, only seldom in association with functional fission events [35, 36]. At the functional division sites on mitochondrial tubules, Dnm1p self-assembles further into spiral-like structures around constricted mitochondrial tubules to promote mitochondrial division upon GTP hydrolysis [37–39].

#### Fis1 and Mdv1p

Further genetic approaches in yeast identified Fis1p and Mdv1p as essential for Dnm1p-mediated mitochondrial fission, and either *fis1* or *mdv1* mutations inhibit fission, resulting in mitochondrial elongation [40–43]. Fis1p is a small tail-anchored mitochondrial outer membrane protein with its N-terminal region facing the cytoplasm. When ectopically expressed Fis1p is evenly distributed in the outer membrane of mitochondria [41]. Fis1p is required for assembly and distribution of Dnm1p on the surface of mitochondria and it acts to coordinate the recruitment and assembly of cytosolic Dnm1p on mitochondria by interaction with Dnm1p through one of two adaptor proteins, Mdv1p or its paralog Caf4p [34, 40, 41, 44]. It is generally believed that Fis1p functions as a mitochondrial receptor to recruit cytosolic Dnm1p to the mitochondrial surface. However, the molecular mechanism by which Dnm1p is recruited to the punctate structures on the surface of mitochondria is still poorly understood [45, 46], as the suggested receptor Fis1p is evenly localized on the surface of mitochondria [41].

In the absence of Fis1p alone or both Fis1p and Mdv1p, the Dnm1p-containing punctate structures are reduced in number along mitochondrial tubules, but Dnm1p is still able to assemble into a few large punctate structures on the mitochondrial membrane [34, 35, 40, 41, 46]. However, these Dnm1p-containing structures lacking Fis1p or Mdv1p are abnormal and unable to mediate mitochondrial fission [40, 41]. This indicates that there may be additional unknown molecular mechanisms responsible for the recruitment of cytosolic Dnm1p to discrete sites on mitochondria in *fis1*∆ or *fis1*∆ *mdv1*∆ yeast cells [40, 41].

**Fig. 2:**
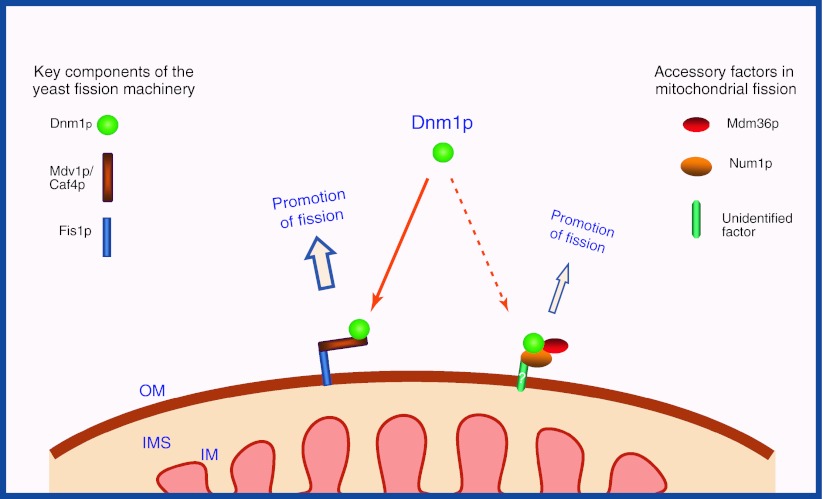
A model for regulation of mitochondrial fission in yeast. The mitochondrial outer membrane-anchored protein Fis1p serves as a key mitochondrial receptor to initially recruit the adaptor Mdv1p or Caf4p to the surface of mitochondria. The Fis1p–Mdv1p/Caf4p complex recruits and assembles cytosolic Dnm1p to mitochondrial division sites to drive mitochondrial fission. In addition to the major fission pathway, Num1p and Mdm36p are proposed to recruit Dnm1p to mitochondria via an unidentified mitochondrial outer membrane protein to trigger mitochondrial fission. *OM* outer membrane, *IM* inner membrane, *IMS* intermembrane space

Mdv1p is a soluble cytosolic protein containing an N-terminal extension (NTE), a middle coiled-coil (C–C) domain and a C-terminal WD repeat (WD), and is peripherally associated with the mitochondrial outer membrane [40, 42, 43]. As a molecular bridge between Fis1p and Dnm1p, Mdv1p binds to Fis1p through the N-terminal extension and to Dnm1p through the C-terminal WD repeat (Fig. 2), while the central coiled-coil domain of Mdv1p mediates homo-oligomer formation [44, 46]. Thus, Mdv1p serves as an adaptor providing a connection between Fis1p and Dnm1p during mitochondrial division [47]. Mdv1p is present in punctate structures and co-localizes with Dnm1p on the mitochondrial outer membrane [38, 40, 43].

When Dnm1p is absent, Mdv1p (complexed with Fis1p) is uniformly distributed along the outer mitochondrial membrane and fails to form punctate structures [34, 38, 40, 43]. Also, when the Dnm1p–Mdv1p interaction is disrupted by either a mutation in the WD-repeats of Mdv1p or a defect in the GTPase region of Dnm1p, Mdv1p is found to be evenly localized along the mitochondrial surface [46]. These data suggest that Dnm1p plays an essential role in inducing the formation of the Mdv1p punctate structures [45]. Moreover, in cells lacking Mdv1p, Dnm1p is still localized to punctate structures along the mitochondrial membrane similar to in wild-type cells, but these Dnm1p-containing structures lacking Mdv1p on mitochondria are unable to complete division [40, 42, 43]. This suggests that Mdv1p is not required for the recruitment of Dnm1p to the mitochondrial membrane or for Dnm1p assembly into punctate structures [45], but that Mdv1p is required for normal Dnm1p function. Furthermore, in cells lacking Fis1p, most of Mdv1p is retained in abnormal punctate structures with Dnm1p on mitochondria and only a small fraction of Mdv1p is in the cytoplasm [40], indicating that Fis1p is required for the normal assembly and/or distribution of Dnm1p/Mdv1p-containing complexes on the mitochondrial membrane. In contrast, in cells lacking both Fis1p and Dnm1p, Mdv1p is cytoplasmic [40, 41], indicating that both Fis1p and Dnm1p are required for the mitochondrial recruitment of Mdv1p [45]. Thus, all three proteins, Dnm1p, Fis1p and Mdv1p work together to drive the normal mitochondrial fission process in yeast.

#### Caf4p

Caf4p is an additional component of the mitochondrial fission machinery in yeast and was identified by affinity purification and mass spectrometry as a binding partner of Fis1p. Caf4p is structurally similar to Mdv1p, with an N-terminal extension, a central coiled-coil domain, and a C-terminal WD repeat, and is peripherally associated with the mitochondrial outer membrane in a Fis1p-dependent manner. Caf4p binds to Fis1p, Dnm1p, and Mdv1p [34]. In the absence of Mdv1p, the Fis1p-Caf4p complex is able to recruit Dnm1p to the mitochondrial membrane, thus acting in a similar manner to Mdv1p as a molecular adaptor to recruit Dnm1p to mitochondria, and promote mitochondrial fission. However, Caf4p is not essential for mitochondrial division as in cells lacking Caf4p, mitochondrial morphology is indistinguishable from wild-type [34].

Deletion of Mdv1p or Caf4p alone has only a modest effect on the mitochondrial localization of Dnm1p [34, 35, 40]. In contrast, in *mdv1∆* *caf4∆* cells, the punctate structures of Dnm1p along mitochondrial tubules are reduced, although a few large punctate Dnm1p structures do still localize to mitochondria [34], which is similar to the *fis1∆* cells [40, 41]. Thus, it could not be excluded that additional factors besides Fis1p and Mdv1p/Caf4p are involved in the recruitment of Dnm1p to the surface of mitochondria in yeast.

### Potential players and co-factors in the yeast fission machinery

#### Num1p

Num1p (nuclear migration 1) is a large cell cortex-anchored protein involved in nuclear segregation. A genome-wide screen of yeast deletion mutants showed that *Num1* has a role in maintaining mitochondrial dynamics [48]. *Num1* mutant yeast cells contain an interconnected network of mitochondrial tubules, similar to cells lacking Dnm1p. Num1p normally assembles into punctate structures on mitochondria, which colocalize with a subset of mitochondria-bound Dnm1p complexes. Interestingly, Num1p interacts with Dnm1p but not with Mdv1p, and cells lacking Num1p show an increase of Dnm1p in the cytosol, suggesting that Num1p may play a role in the recruitment of Dnm1p to mitochondria or in maintaining the stability of Dnm1p on the mitochondrial surface. Num1p binds to mitochondria independent of Fis1p, Mdv1p and Dnm1p. It was therefore suggested that Num1p provides an additional attachment site for Dnm1p on mitochondria. However, ~50 % of Dnm1p-GFP remains bound to mitochondria in cells lacking both Num1p and Fis1p, indicating that there are still additional unknown factors involved in recruiting Dnm1p to mitochondria besides Fis1p and Num1p in yeast. Although Num1p has a role in mitochondrial division in budding yeast, it is not essential for the yeast fission machinery as *num1*Δ mutants retain some fission ability [49].

#### Mdm36p

The MDM36 gene was discovered by screening yeast deletion mutants for aberrant mitochondrial distribution and morphology [48]. A recent study shows that yeast cells lacking Mdm36p contain highly interconnected mitochondrial networks that resemble the phenotype of known fission mutants. At the same time, the colocalization of Num1p and Dnm1p is abolished in these yeast cells, suggesting that Mdm36p is required for the formation of Num1p-Dnm1p complexes. Mdm36p is associated with mitochondria by an as yet unknown mechanism and plays an important role in the attachment of mitochondria to the cell cortex via Dnm1p and Num1p. Mdm36p and Num1p might act in the same cellular pathway. Like Num1p, Mdm36p is suggested to act as an accessory component rather than an essential part of the mitochondrial division machinery [50].

#### Mdm33p

Mdm33p contains two transmembrane segments and is an integral mitochondrial inner membrane protein. Yeast cells lacking Mdm33p contain ring-shaped, mostly interconnected mitochondria and the phenotype of the *mdm33*Δ mutant resembles other yeast mutants affecting mitochondrial fission, such as *dnm1*Δ, *mdv1*Δ, and *fis1*Δ. Over-expression of Mdm33p leads to cell growth arrest, aggregation of mitochondria, and generation of aberrant inner membrane structures, including septa, inner membrane fragments, and loss of inner membrane cristae. Thus, Mdm33p has been proposed to be involved in fission of the mitochondrial inner membrane [51]. This report raises the issue whether mitochondrial fission requires the integrated, balanced fission of both the outer and inner mitochondrial membranes.

### Models for mitochondrial fission in yeast

Yeast mitochondrial fission is believed to be a multistep process, during which Fis1p initially recruits Mdv1p to the mitochondrial membrane, and this Fis1p-Mdv1p complex mediates recruitment and assembly of Dnm1p in punctate structures along mitochondrial tubules at constriction sites. At the constriction sites Dnm1p further forms into multimeric structures by self-interaction. The GTPase activity of Dnm1p is ultimately essential for driving mitochondrial division (Fig. 2). Dnm1p-dependent mitochondrial fission is therefore regulated by the cytosolic protein Mdv1p and the mitochondrial outer membrane protein Fis1p. Mdv1p functions as a molecular adaptor to regulate interactions between Dnm1p and Fis1p, while Fis1p acts as a mitochondrial receptor to regulate the recruitment of Dnm1p to the outer mitochondrial membrane [3, 30].

However, in such a model it is difficult to envisage how Fis1p can recruit Dnm1p to specific division sites because Fis1p is by itself evenly distributed along mitochondrial tubules. An alternative model has been proposed, in which Dnm1p binds first to potential division sites on the mitochondrial surface by as yet unknown molecular mechanisms to induce recruitment of the effector proteins Fis1p and Mdv1p to these sites, where they all assemble into large dot-like structures, and form the fission machinery along mitochondrial tubules. When the fission machinery is stimulated by additional molecular signaling, Dnm1p can further oligomerize to form spiral-like structures around the mitochondrial tubules to drive mitochondrial division [46].

In addition to the major fission pathway, Num1p and Mdm36p have been proposed to play a role in the recruitment of Dnm1p to mitochondria via an as yet-unidentified, Fis1p-independent mechanism (see also Fig. 2) [49, 50]. However, how Num1p, Mdm36p, and the integral inner membrane protein Mdm33p are involved in these processes is so far unclear.

### The key players in the mammalian mitochondrial fission machinery

It is generally believed that the fundamental mechanisms controlling mitochondrial fission are similar between yeast and mammals. For example, the two key players Drp1 and hFis1 (human Dnm1p and Fis1p ortholog, respectively) of the fission machinery are evolutionarily conserved from yeast to mammals. As discussed above, in yeast, the mitochondrial receptor Fis1p recruits Dnm1p to the mitochondrial outer membrane through one of the adaptors Mdv1p or Caf4p, but no orthologs of Mdv1p and Caf4p have been identified in mammals. hFis1 is believed to be involved in recruiting Drp1 to mitochondria as in yeast by a direct or indirect interaction with Drp1, possibly through an unknown Mdv1p-like adaptor. In the classical model of the mitochondrial fission machinery in mammals, just as in yeast, Drp1 and hFis1 were proposed to be the two core components of the fission machinery. During mitochondrial fission, the mitochondrial outer membrane protein hFis1 was proposed to act as a mitochondrial receptor to recruit cytoplasmic Drp1 to potential division sites along the mitochondrial surface, where it is assembled into a higher-order complex. Drp1 is thought to wrap around the mitochondria to induce mitochondrial fission via its GTPase activity [12, 30].

#### Drp1

Drp1 plays a central role in mitochondrial fission also in mammals. One study has shown that an infant born with a dominant negative Drp1 mutation that resulted in a severely defective mitochondrial fission process, displayed microcephaly, abnormal brain development and metabolic aberrations and died at 37 days of age [17]. Recent studies from mouse knockout models indicate that Drp1 is essential for mouse embryonic and brain development and mice lacking Drp1 die at an early embryonic stage [15, 16]. Drp1, like its yeast ortholog Dnm1p, is a dynamin-related GTPase and is primarily distributed in the cytoplasm. But a fraction of Drp1 localizes to dot-like structures on the mitochondrial surface and the protein shuttles between the cytoplasm and mitochondria. To mediate mitochondrial fission, Drp1 must be recruited from the cytoplasm to the mitochondrial surface. Depletion of Drp1 either by siRNA or by overexpressing a dominant negative mutant Drp1^K38A^ leads to elongated inter-connected tubular networks of mitochondria. The GTPase activity of Drp1 is essential for Drp1-mediated mitochondrial fission [12, 52, 53].

#### hFis1

hFis1 (human Fis1) is a C-terminal anchored mitochondrial outer membrane protein with its N-terminal part exposed to the cytosol. Like its yeast homologue Fis1p, hFis1 is evenly distributed on the mitochondrial surface [54–56]. Over-expression of hFis1 induces mitochondrial fragmentation. Inhibition of hFis1 function results in mitochondrial elongation, thus mammalian hFis1 was and still is proposed to have a role similar to its yeast homologue Fis1p [54–56]. Although homologues of the yeast adaptor proteins Mdv1p and Caf4p that interact with both Dnm1p and Fis1p have not been found in mammals, a weak and transient interaction was observed between hFis1 and Drp1 [54, 57]. Therefore, hFis1 was suggested to serve as a potential receptor for the recruitment of cytoplasmic Drp1 to the mitochondrial surface in mammals.

However, the view that hFis1 is a *bona fide* mitochondrial receptor for Drp1 in mammals has been challenged. First, no orthologs of Mdv1p and Caf4p have been found in mammals although these proteins are required for bridging between Dnm1p and Fis1p in yeast. Second, hFis1 is uniformly distributed throughout the mitochondrial outer membrane [55, 56, 58], while Drp1 is localized to punctate structures along the mitochondrial tubules [52]. It is difficult to imagine how Drp1 can be recruited to punctate division sites on the mitochondrial surface through hFis1 if this is to occur without additional targeting signals on mitochondria. Third, increased or reduced levels of hFis1 do not affect the distribution of or amount of Drp1 along mitochondria [58–60]. Moreover, hFis1 deficient human cells still maintain a normal mitochondrial morphology and fission-competent mitochondria, and the recruitment of Drp1 to mitochondria is not affected [60]. Finally, human hFis1 and yeast Fis1p are not functionally interchangeable in vivo because hFis1 cannot rescue the mutant phenotype observed in *fis1*∆ yeast cells [55], indicating that the two proteins are at least to some extent functionally distinct. Taken together, these data strongly suggested there are additional proteins or other molecular signals on the mitochondrial surface that potentially contribute to the recruitment of Drp1 to the mitochondrial outer membrane in mammals. However, it should be stressed that hFis1 might have a crucial role in the recruitment of Drp1 to mitochondria under certain conditions including cell stress-induced mitochondrial fission. For example, an increased interaction of Fis1 with Drp1 and recruitment of Drp1 to mitochondria was observed in hypoxia-mediated mitochondrial fission in a recent study, and reduced expression of Fis1 significantly attenuated hypoxia-mediated mitochondrial fragmentation in mammalian cells [61]. Interestingly, Kim et al. [61] found that the ubiquitin ligases Siah1a/2 are required for hypoxia-mediated mitochondrial fission through controlling the expression levels of AKAP121, a Siah2 substrate, to affect the level of Fis1–Drp1 interaction and mitochondrial dynamics under hypoxia. Although the underlying mechanisms remain elusive, over-expression of AKAP121 reduced the interaction of Drp1 with Fis1, while knock-down of AKAP121 by shRNA increased Drp1–Fis1 interaction [61]. This suggested that Fis1 is required for translocation of Drp1 to mitochondria in response to hypoxia-triggered mitochondrial fission. Collectively, elevated levels of Siah1a/2 under hypoxia lead to decreased levels of AKAP121, in turn resulting in an increased Drp1–Fis1 interaction and ultimately mitochondrial fission.

Recently, several reports have shown that Drp1 is recruited from the cytoplasm to the mitochondrial surface through an increased interaction between Drp1 and hFis1, and that mitochondria undergo rapid and excessive fission when human and other mammalian cells are treated with a variety of apoptotic stimuli [62–64]. These findings suggested that although the basal level of interaction between Drp1 and hFis1 is usually very low in healthy cells, a significant proportion of Drp1 becomes associated with hFis1 in cells treated with apoptotic stimuli [62–64]. Thus, hFis1 may play an important role in the recruitment of Drp1 to mitochondria during apoptotic stimuli-induced mitochondrial fission.

#### Mff

An important Drp1-recruiting factor, the mitochondrial fission factor (Mff), has recently been identified [60, 65]. Mff is a mitochondrial receptor of Drp1, conserved in metazoans but not in yeast. Mff was first discovered by high-throughput screening of a *Drosophila* RNA interference (RNAi) library for mitochondrial morphology alterations. The silencing of one gene, called CG30403/Tango11, induced a phenotype with perinuclear clustering of mitochondria similar to that in cells depleted of Drp1. The ortholog protein in human was named mitochondrial fission factor (Mff) [65]. Mff is anchored to the mitochondrial outer membrane through a C-terminal transmembrane domain. The bulk of the protein including the N-terminus is exposed to the cytosol. Depletion of Mff by siRNA in mammalian cells promotes mitochondrial fusion, resulting in an interconnected tubular network of mitochondria, similar to the phenotype in cells depleted of Drp1. In contrast, exogenous expression of Mff induces extensive mitochondrial fragmentation. Moreover, Mff and hFis1 are present in separate complexes, suggesting that they play distinct roles in mitochondrial fission [65].

More recently, Otera et al. found that Mff is localized in punctate structures on mitochondria in a manner independent of Drp1 and hFis1, and in contrast to the uniform distribution of hFis1 on the mitochondrial outer membrane. Mff mostly co-localizes with Drp1 in these dot-like structures along the mitochondrial tubules. Furthermore, Mff was shown to be able to interact with Drp1 through its N-terminal region. Over-expression of Mff promotes Drp1’s mitochondrial association and is accompanied by mitochondrial fragmentation. In contrast, knock-down of Mff by siRNA reduces the recruitment of Drp1 to mitochondria, resulting in mitochondrial elongation. Finally, Otera et al. showed that Mff-mediated mitochondrial fission is independent of hFis1. Conditional knock-down of hFis1 in colon carcinoma cells revealed that hFis1 did not affect mitochondrial fission. Taken together, these observations indicate that Mff functions as a mitochondrial receptor for the recruitment of Drp1 to the mitochondrial surface thereby driving Drp1-dependent mitochondrial division in metazoans [60, 66]. The characterization of Mff also suggests that the molecular mechanisms of the mitochondrial fission machinery evolutionarily are distinct in metazoans as compared to yeast. Moreover, the Mff gene encodes at least nine different isoforms generated by alternative splicing in human [65], indicating that the regulation of Drp1’s recruitment to mitochondria is likely more complex in human although the functional characteristics of these Mff isoforms remain to be analyzed. It will be interesting to learn whether Mff also plays a role in the later steps of the Drp1-mediated fission process.

#### MIEF1, a novel vertebrate-specific regulator of mitochondrial fission

Mitochondrial elongation factor 1 (MIEF1) was discovered by searching an intracellular protein localization database that contains a large number of green fluorescent protein (GFP)-tagged fusion proteins from human [67]. MIEF1 is encoded by a gene originally named *SMCR7L* (Smith–Magenis syndrome chromosomal region candidate gene 7 protein-like) on chromosome 22, and the gene product was designated as MIEF1 (Mitochondrial elongation factor 1) as its expression leads to an extensive mitochondrial elongation [68]. MIEF1 is an integral mitochondrial outer membrane protein anchored through an N-terminal transmembrane domain with its C-terminal major part exposed to the cytoplasm. The protein is highly conserved in all vertebrate species analyzed, but is not found in yeast, invertebrates and plants [68]. Ectopic expression of MIEF1 increases mitochondrial fusion leading to extensively elongated mitochondria, whereas depletion of MIEF1 enhances mitochondrial fragmentation, indicating that MIEF1 plays a role in inhibiting mitochondrial fission or/and promoting fusion.

**Fig. 3:**
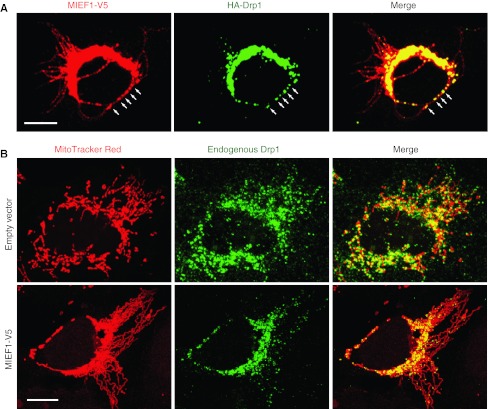
Over-expression of MIEF1 recruits cytosolic Drp1 to the surface of mitochondria and promotes mitochondrial fusion rather than fission. **a** Confocal images showing that introduced MIEF1-V5 co-localizes with introduced HA-Drp1 in punctate structures (*arrows*) along the mitochondrial tubules. **b** Mitochondrial morphology and the distribution of endogenous Drp1 in 293T cells transfected with either empty vector (*upper panel*) or MIEF1-V5 plasmid (*lower panel*). *Bars* represent 10 μm

It is generally believed that during mitochondrial fission, Drp1 is actively recruited to the mitochondrial outer membrane. It was found that MIEF1 is co-localized with Drp1 in the punctate structures along the mitochondrial surface (Fig. 3a). Co-immunoprecipitation revealed that MIEF1 forms complexes with endogenous Drp1. Over-expression of MIEF1 increased the recruitment of cytosolic Drp1 to the mitochondrial surface (Fig. 3b) and silencing of the fission proteins hFis1 and Mff and the fusion protein Mfn2 by siRNA did not affect the MIEF1-mediated recruitment of Drp1 to mitochondria [68]. These data indicated that MIEF1 is a mitochondrial receptor for the recruitment of cytosolic Drp1 to mitochondria in a manner independent of hFis1, Mff and Mfn2. Importantly, it was observed that MIEF1 binding to Drp1 and the MIEF1-mediated recruitment of Drp1 to mitochondria were independent of Drp1’s GTPase activity or phosphorylation status. MIEF1 can efficiently bind to a dominant negative Drp1^K38A^ mutant (lacking the GTPase activity), the phosphorylation-deficient Drp1^S637A^ mutant and the phosphomimetic Drp1^S637D^ mutant and recruits all these proteins to mitochondria [68].

Despite the fact that MIEF1 recruits Drp1 to mitochondria, it promotes mitochondrial fusion rather than fission (Fig. 3b). It has therefore been proposed that MIEF1 acts as a suppressor of Drp1 function by sequestering the protein on the mitochondrial outer membrane and inhibiting its activity. MIEF1’s negative regulatory role in mitochondrial fission therefore results in a fusion phenotype [68, 69]. Several lines of additional evidence support that MIEF1 inhibits Drp1 activity. Firstly, MIEF1 over-expression reduces the GTP-binding levels of endogenous Drp1, suggesting that it may affect the GTPase activity of Drp1 via a reduction of its GTP-binding. Secondly, analysis of a set of MIEF1 deletion mutants revealed that all MIEF1 mutants that retain the ability to bind Drp1 cause a mitochondrial fusion phenotype, whereas a MIEF1^∆160–169^ mutant lacking Drp1-binding does not promote fusion. Thirdly, the deletion mutant MIEF1^∆1–48^ lacking the transmembrane region is diffusely distributed in the cytoplasm, while retaining its ability to bind Drp1. Moreover, the MIEF1^∆1–48^ mutant sequesters Drp1 in the cytoplasm, thereby inducing a mitochondrial fusion phenotype that is similar to those induced by a dominant negative Drp1^K38A^ mutant or by knock-down of either Drp1 or Mff [68]. Collectively, these data indicated that inhibition of Drp1 function via a MIEF1–Drp1 interaction is the key mechanism for the MIEF1-induced mitochondrial fusion phenotype.

Intriguingly, co-immunoprecipitation showed that MIEF1 also associates with hFis1 in a manner independent of its Drp1 binding, and MIEF1 binding to Drp1 and hFis1 occurs in the form of two separate complexes in a mutually exclusive manner. Increased levels of hFis1 can partially reverse the MIEF1-induced fusion phenotype. It was proposed that the hFis1–MIEF1 interaction might prevent MIEF1 from acting as a fission inhibitor by sequestering Drp1. These findings of mutually exclusive MIEF1–Drp1 versus MIEF1–hFis1 interactions suggest a novel key regulatory mechanism for mitochondrial dynamics in mammals.

Notably, MIEF1 also known as MiD51 and its paralog MiD49 (mitochondrial dynamics proteins of 51 and 49 kDa, respectively) were independently identified in a recent report [69]. MiD49 is encoded by a gene originally named *SMCR7* (Smith–Magenis syndrome chromosomal region candidate gene 7) on chromosome 17. MIEF1/MiD51 and MiD49 share 45 % identity at the amino acid sequence level. Like MIEF1/MiD51, Palmer et al. [69] found that overexpression of MiD49 interacts with and recruits Drp1 to mitochondria, resulting in mitochondrial elongation. However, some discrepancies were observed in the two studies by Zhao et al. [68] and Palmer et al. [69], upon depletion of MIEF1/MiD51 and MiD49. Zhao et al. [68] observed that knockdown of MIEF1/MiD51 alone enhances mitochondrial fragmentation, whereas Palmer et al. [69] found that depletion of MIEF1/MiD51 or MiD49 alone does not affect mitochondrial morphology, but depletion of the two proteins causes mitochondrial elongation. Although the cause of these discrepancies between the two studies is currently not clear, they are probably due to differences used in the knockdown experiments, for instance the choice of cell lines, selection of siRNAs and levels of the endogenous targeted proteins. However, it is clear that further studies will be required to address this issue.

### A modified molecular model for the mitochondrial fission machinery in mammals

**Fig. 4:**
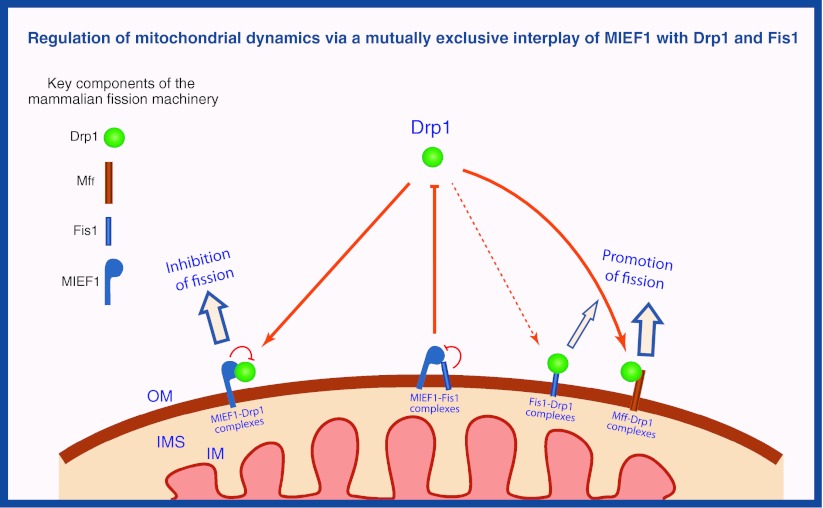
A model for regulation of mitochondrial fission in vertebrates. Three mitochondrial outer membrane-anchored proteins Fis1, Mff and MIEF1 serve as mitochondrial receptors to recruit cytosolic Drp1 to the surface of mitochondria. Under normal conditions, Mff forms complexes with Drp1 to promote mitochondrial fission, but in some conditions Fis1 can also form complexes with Drp1 to trigger mitochondrial fission, such as in cell stress- and hypoxia-mediated mitochondrial fission. Conversely, MIEF1–Drp1 complexes sequester Drp1 and inhibit Drp1-driven mitochondrial fission. MIEF1 also forms complexes with Fis1, which impedes complex formation between MIEF1 and Drp1, thereby relieving MIEF1’s inhibitory effect on Drp1. *OM* outer membrane, *IM* inner membrane, *IMS* intermembrane space

Taking these recent studies together with previous data, a modified model for the regulation of Drp1-mediated mitochondrial fission can be proposed for vertebrates (Fig. 4). In this model, three integral mitochondrial outer membrane proteins each with a single TM domain, hFis1, Mff and MIEF1 are suggested as potential receptors for recruitment of cytosolic Drp1 to the mitochondrial surface, but the three proteins are thought to have distinct roles in regulating Drp1-mediated mitochondrial fission. The recruitment of Drp1 by Mff promotes mitochondrial fission. In contrast, Drp1 binding to MIEF1 sequesters Drp1 at the mitochondrial surface, inhibits Drp1 activity by reducing its GTP binding and promotes mitochondrial fusion. In the previous model, mammalian hFis1 was, like its ortholog Fis1p in yeast, considered to act as a key receptor for Drp1, but its function has been challenged recently. However, in some conditions hFis1 can also form complexes with Drp1 to trigger mitochondrial fission, such as cell stress- and hypoxia-mediated mitochondrial fission. Alternatively, we have found that hFis1 can form a complex with MIEF1 in a mutually exclusive manner to the Drp1–MIEF1 complex [68]. In that way, hFis1 is suggested to play an additional role in reducing the inhibitory effect of MIEF1 on Drp1 activity. By formation of an hFis1-MIEF1 complex, MIEF1 binding to Drp1 will be prevented and this will allow for mitochondrial fission to occur.

The suggested model (Fig. 4) highlights that the molecular machinery for controlling mitochondrial dynamics has evolved such that two of the central components, the Dnm1p/Drp1 and Fis1p/hFis1 proteins, are highly conserved in both yeast and vertebrates, while their interacting proteins, i.e., Mdv1p and Caf4p in yeast, Mff and MIEF1 in vertebrates, are quite evolutionarily and functionally diverged. The hFis1–Drp1 interaction is no longer a unique pathway responsible for Drp1 recruitment to mitochondria in higher eukaryotes. One or more additional pathways, highlighted by the novel factors Mff and MIEF1, have emerged in mammals for a fine-tuned regulation of Drp1 recruitment and mitochondrial fission. Overall, these recent studies have shed light on the longstanding question of how Drp1 is recruited to the mitochondrial membrane and how Drp1-mediated fission is regulated in vertebrates. However, many questions are still waiting to be elucidated, e.g., how Mff and MIEF1 coordinate Drp1’s function at the mitochondrial surface and whether these two factors are co-localized in the same punctate structures along mitochondrial tubules and/or may belong to two different pathways of Drp1 recruitment.

### Other potential players and co-factors in the mammalian mitochondrial fission machinery

Besides Drp1, hFis1 and the two newly identified key factors Mff and MIEF1, several additional proteins have been proposed to regulate mitochondrial fission in mammals, including endophilin B1 [70], MTP18 (mitochondrial protein 18 kDa) [71, 72], GDAP1 (ganglioside-induced differentiation-associated protein 1) [73] and MTGM (mitochondrial targeting GxxxG motif protein) [74]. Endophilin B1 is primarily present in the cytosol, and only a small fraction localizes to mitochondria. The protein cycles dynamically between the cytosol and the outer mitochondrial membrane. Depletion of the protein affects the shape of mitochondria [70]. Endophilin B1 is proposed to act in a pathway downstream of Drp1, but the molecular mechanisms mediated by the protein remain to be elucidated. GDAP1 is an integral mitochondrial outer-membrane protein [73], MTP18 is located in the intermembrane space and is an inner membrane-associated protein [71, 72], and MTGM is an integral inner membrane protein [74]. These three proteins have been implicated in the regulation of Drp1-dependent mitochondrial fission because their over-expression causes mitochondrial fragmentation, whereas their knock-down leads to mitochondrial elongation [71–74]. However, whether these proteins are directly involved in the “classical” mitochondrial fission pathway and how they co-ordinate their actions with the key proteins Drp1, Mff, hFis1 and MIEF1 during mitochondrial fission is currently poorly understood.

Notably, a co-ordination of the mitochondrial outer and inner membranes is believed to be required for mitochondrial division. However, the crucial inner membrane proteins that are involved in the fission process remain to be elucidated [10]. Two potential candidates that might participate in co-ordinated fission of the mitochondrial inner membrane have been proposed in the literature, including MTP18 and MTGM [71, 72, 74]. MTGM is a small protein of 79 amino acids that is anchored in the inner membrane by a single TM domain located in the middle of the protein. The protein is highly conserved from yeast to human with 100 % identity in mammalian species. Ectopic expression of MTGM triggers mitochondrial fragmentation and knock-down of MTGM by RNAi induces mitochondrial elongation [74]. Although there is a predicted MTGM homologue encoded by the gene known as MGR2 in yeast, it is unknown whether the potential yeast homologue affects mitochondrial dynamics in yeast as well. It will be interesting to learn if and how the actions of MTP18 and MTGM may be coordinated with the key proteins involved in promoting/inhibiting outer membrane fission, such as Mff, hFis1, MIEF1 and Drp1.

### Regulatory mechanisms of the mitochondrial fission machinery in mammals

Regulation of the mitochondrial fission machinery through post-translational modifications of Drp1 has been reported in mammals. Drp1 is post-translationally modified in multiple ways and these modifications can in turn be regulated by different signaling pathways in the cells. Post-translational modifications of Drp1, including protein phosphorylation, sumoylation, ubiquitination, and *S*-nitrosylation, have been implicated in regulating Drp1-mediated mitochondrial fission [75, 76]. Cdk/cyclin B [77], PKA [78, 79] and CaMKIα [80] are involved in phosphorylation, and the phosphatase calcineurin (PP2B) [78, 81] in dephosphorylation of Drp1; SUMO1 [82], MAPL [83] and SENP5 [84] are involved in the positive and negative regulation of Drp1 sumoylation; MARCH5/MARCH-V/MITOL [85–88] and Parkin [89] are involved in Drp1 ubiquitination; and nitric oxide (NO) is involved in Drp1 *S*-nitrosylation [90]. In addition, microRNA, for instance miR-499, has been recently reported to modulate calcineurin-mediated dephosphorylation of Drp1 by targeting calcineurin [91]. Potential roles of these post-translational modifications in the regulation of mitochondrial dynamics have been presented in several recent reviews [75, 76]. Here, we present the proteins involved in modifying components of the mitochondrial fission machinery in Table 1 as a comparison between yeast and mammals. It can be seen from this table that most of the proteins involved in Drp1 modifications in mammals have not been found in yeast. Although the potential roles and mechanisms of these post-translational modifications affecting Drp1-driven mitochondrial fission are not currently fully understood, a growing body of evidence indicates that regulation of mitochondrial dynamics through various modifications of pro-fission proteins, especially Drp1, has evolved to become considerably more elaborate in mammals than in yeast. Besides Drp1, little is known about post-translational modifications of other key fission-promoting proteins. It is highly possible that these types of modifications contribute an additional layer of mechanisms for fine-tuning the mitochondrial fission process in mammals.

## Mitochondrial fusion in yeast and vertebrates

### The key players of the mitochondrial fusion machinery in yeast

**Fig. 5:**
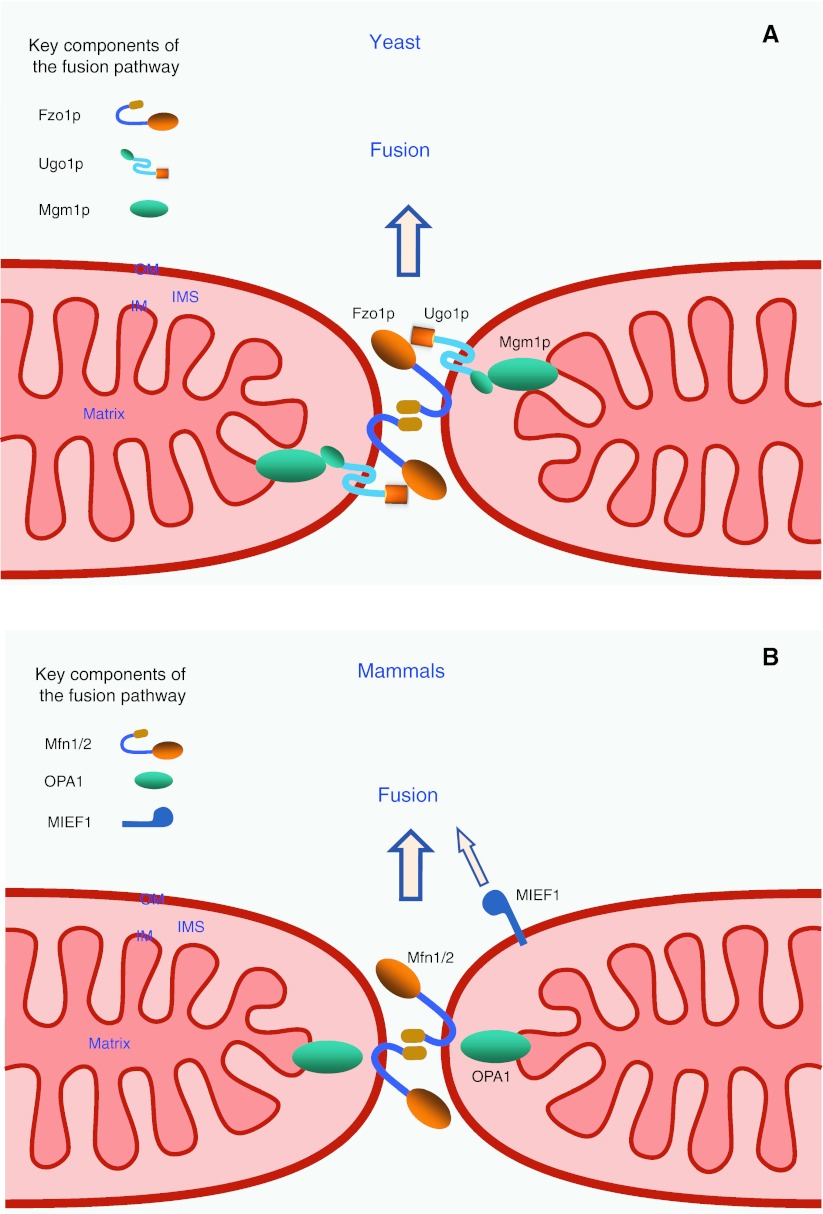
The mitochondrial fusion machineries in yeast and mammals. **a** A model for the mitochondrial fusion events in yeast. Adjacent mitochondria are tethered through the formation of Fzo1p *trans* complexes to promote fusion of the mitochondrial outer membranes (OM). Subsequently, Mgm1p is involved in tethering inner membranes together to promote fusion of the inner membranes (IM). Ugo1p is proposed to play an important role in coordinating outer and inner membrane fusion events. **b** In mammals, there are two orthologs Mfn1 and Mfn2 of yeast Fzo1p. Mfn1 and Mfn2 interact with each other to coordinate tethering and fusion of the outer membrane of adjacent mitochondria, and OPA1 (ortholog of yeast Mgm1p) is essential for fusion of the inner membrane. In addition, MIEF1 is also proposed to promote mitochondrial fusion in a manner that does not require Mfn2. *IMS* intermembrane space

The mitochondrial fusion machinery has been well-characterized in *S. cerevisiae*. Mitochondrial fusion requires at least three core components in yeast, two outer membrane-anchored proteins, Fzo1p (a GTPase) and Ugo1p, and one inner membrane-anchored GTPase Mgm1p. These three proteins form protein complexes that mediate mitochondrial fusion in yeast and cells lacking any of these proteins show fragmented mitochondria [2, 3, 29, 30]. During mitochondrial fusion, Fzo1p molecules on adjacent mitochondria form *trans* complexes via interaction of their C-terminal coiled-coil region tethering the outer membranes of mitochondria together, and resulting in outer membrane fusion of adjacent mitochondria via the GTPase activity of Fzo1p. Similarly, Mgm1p is able to form *trans* complexes to tether apposing inner membranes together, leading to inner membrane fusion via Mgm1p GTP hydrolysis. Ugo1p interacts with both Fzo1p and Mgm1p to form a complex, which is believed to be vitally important in co-ordinating outer and inner membrane fusion events in yeast (Fig. 5).

#### Fzo1p (Mfn1/2 in mammals)

Yeast *FZO1* [92, 93] was identified as a homologue of the *fuzzy onions* (Fzo) gene in *Drosophila* [94]. The gene is highly conserved from yeast to human. Mammalian genomes contain two Fzo homologues, the mitofusins, Mfn1 and Mfn2 [13, 95]. Yeast Fzo1p is a large GTPase anchored in the mitochondrial outer membrane by two adjacent transmembrane segments close to the C-terminus, with a highly conserved N-terminal GTPase domain, and four putative heptad repeats (coiled-coil domains) [94], a short loop between the two transmembrane segments exposed to the intermembrane space [96] and both the N- and C-termini exposed to the cytosol, mediating inter-mitochondrial interactions [92, 93, 97]. Fzo1p is required for maintaining the tubular mitochondrial reticulum and plays an essential and direct role in mitochondrial fusion during yeast mating [93]. Absence of the FZO1 gene in yeast results in fragmented mitochondria, loss of mtDNA, lack of inner membrane cristae and mitochondrial clustering [92, 93]. A recent study shows that Fzo1p can assemble into a homo-dimer in the outer membrane of mitochondria mediating mitochondrial tethering and fusion. This Fzo1p dimerization depends on its GTP binding and on its interaction with Ugo1p [98].

#### Mgm1p (OPA1 in mammals)

*MGM1* was first discovered as a gene involved in the maintenance of the mitochondrial genome in a screen for yeast mutants [99]. Like Fzo1p, Mgm1p is also conserved from yeast to human. Mgm1p is a dynamin-related GTPase, essential for mitochondrial inner membrane fusion. It contains an N-terminal mitochondrial targeting sequence that is cleaved by matrix-processing peptidase (MPP) following import and the protein is anchored to the inner membrane through an N-terminal transmembrane domain. The bulk of the protein including the GTPase domain, a middle domain and two hydrophobic segments is exposed in the intermembrane space [100–102]. Mgm1p exists in two forms at steady state, a short isoform is located in the intermembrane space, and a large isoform is inserted in the inner membrane by its N-terminal transmembrane domain. The short form lacks the transmembrane anchor and is produced through the cleavage of the large form of Mgm1p by the rhomboid-related membrane protease Pcp1p [103, 104]. Both the long and short isoforms are necessary for efficient mitochondrial fusion to occur but not essential for fusion to occur at all [103–105]. Mgm1p mutations cause mitochondrial fragmentation and aggregation, and loss of mtDNA [101, 106]. Mgm1p is associated with the two outer membrane-anchored fusion-promoting proteins Fzo1p and Ugo1p in mitochondria, thus it plays a role in coordination between the inner membrane and outer membrane during mitochondrial fusion [102].

#### Ugo1p

*UGO1* was first isolated in a screen of yeast mutants that lose mtDNA in a Dnm1p-dependent manner [107]. Functional defects of Ugo1p cause mitochondrial fragmentation, loss of mtDNA and respiratory defects in yeast [107]. However, potential homologues of Ugo1p have not been identified in vertebrates. Ugo1p is anchored in the mitochondrial outer membrane by three transmembrane segments, with its N-terminus facing the cytosol and C-terminus in the intermembrane space [107–109]. The cytoplasmic domain of Ugo1p interacts directly with Fzo1p, whereas its intermembrane space domain binds to the inner membrane protein Mgm1p [100, 102, 110]. Moreover, Ugo1p is required for interaction between Fzo1p and Mgm1p [110]. Therefore, Ugo1p has been proposed to function as a bridge that connects the outer membrane via Fzo1p with the inner membrane via Mgm1p and coordinates mitochondrial double-membrane fusion in yeast. Ugo1p function is required for both outer and inner membrane fusion events [109], and it is necessary for Fzo1p to assemble into homodimers facilitating mitochondrial tethering [98].

### Other players and regulators of the yeast mitochondrial fusion machinery

#### Mdm30p

Several proteins have been identified as co-factors in regulating the functional activities of the core components, Fzo1p and Mgm1p in the mitochondrial fusion machinery in yeast. Mdm30p is an F-box protein and mainly present in the cytoplasm but also in mitochondria and is required for maintaining fusion-competent mitochondria in yeast [111]. Cells lacking Mdm30p contain highly aggregated or fragmented mitochondria. Mdm30p controls the mitochondrial shape by regulating the steady-state level of Fzo1p [112]. It is a subunit of a SCF (SKP1-CUL1-F-box protein) E3 ubiquitin-protein ligase complex that mediates the ubiquitination and subsequent proteasomal degradation of Fzo1p [113]. Fzo1p ubiquitination and turnover by Mdm30p occurs only after GTP hydrolysis of Fzo1p in the fusion process, which is required to facilitate mitochondrial fusion [98, 113, 114]. Homologues of Mdm30p have not yet been identified in mammals.

#### Pcp1p (Rbd1p/Mdm37p)

Pcp1p (also known as Rbd1p or Mdm37p) is a rhomboid-related serine protease. The protein is an integral mitochondrial inner membrane protein with six predicted transmembrane segments. Pcp1p is responsible for processing the inner membrane fusion-promoting protein Mgm1p, generating the short isoform that is released into the intermembrane space. Mutants lacking Pcp1p are defective in the processing of Mgm1p and produce only the large isoform of Mgm1p, resulting in partially fragmented mitochondria [103–105]. The Pcp1p protein is functionally conserved from yeast to human, and its mammalian homologue PARL (presenilin-associated rhomboid-like protein) rescues the yeast *pcp1∆* mutant [104].

#### Ups1p

Ups1p is conserved throughout eukaryotes. It is peripherally associated with the mitochondrial inner membrane in the intermembrane space. The human homologue of Ups1p, PRELI, can fully replace Ups1p in yeast cells. Ups1p is required for the maintenance of normal mitochondrial morphology and Pcp1p-dependent processing of Mgm1p [115, 116].

### The key players of the mitochondrial fusion machinery in mammals

In mammals, three key dynamin-related GTPases are required for mitochondrial fusion: the outer membrane GTPases mitofusin 1 (Mfn1) and mitofusin 2 (Mfn2) and the inner membrane GTPase OPA1. These three core proteins of the mitochondrial fusion machinery in mammals are evolutionarily conserved from yeast to human [12, 29, 30]. Like in yeast cells, Mfn1 and Mfn2 (orthologs of yeast Fzo1p) are required for fusion of the outer membrane, and OPA1 (ortholog of yeast Mgm1p) for fusion of the inner membrane in mammalian cells (Fig. 5). However, no mammalian ortholog of yeast Ugo1p has yet been identified. Thus, how to coordinate outer and inner mitochondrial membrane fusion events remains to be elucidated in mammals.

#### Mfn1 and Mfn2 (orthologs of yeast Fzo1p)

Two human genes designated Mitofusin 1 and 2 (Mfn1 and Mfn2) were discovered to be orthologs of *Drosophila fzo* [94]. Mfn1 and Mfn2 are similar in their protein structures and functional domains, containing an N-terminal GTPase domain, two heptad-repeat regions (HR1 and HR2) and two transmembrane segments near the C-terminus. The two human proteins are highly homologous in their amino acid sequences with 60 % identity and 77 % similarity to each other, and the most extensive homology is in the GTPase domain, whereas the least conserved regions are in the N- and C-terminal ends [117]. Both Mfn1 and Mfn2 are anchored in the outer mitochondrial membrane by two transmembrane segments. Their N-terminal GTPase domain and HR1 and the C-terminal HR2 region are oriented towards the cytosol with a short loop facing the intermembrane space [97, 117, 118]. Both Mfn1 and Mfn2 can form homo- and hetero-dimers through trans-interactions of the C-terminal HR2 region, which serves to tether the outer membranes between adjacent mitochondria together [13, 97].

Mfn1 and Mfn2 are required for outer-membrane fusion, and *Mfn1/2*-null embryonic fibroblast cell lines (lacking both Mfn1 and 2) display a loss of fusion-competence and a fragmented mitochondrial phenotype [119]. The proteins are also essential for embryonic development, and mice deficient in either Mfn1 or Mfn2 die in midgestation [13]. Studies of embryonic fibroblasts derived from *Mfn1* deficient and *Mfn2* deficient mice indicate that Mfn1 and Mfn2 have both redundant and distinct functions and coordinate to regulate mitochondrial fusion [13, 119]. The fragmented mitochondrial phenotype in either *Mfn1*-null, *Mfn2*-null or *Mfn1/2*-null cells can be restored by over-expressing either Mfn1 or Mfn2 [13, 97], indicating that a single Mfn is sufficient for inducing mitochondrial fusion [1, 120]. Although the two proteins might play a similar role in mitochondrial fusion, there are functional differences between Mfn1 and Mfn2 [121]. In embryonic fibroblasts, loss of Mfn1 results in a greater degree of mitochondrial fragmentation than loss of Mfn2 [13, 119]. In agreement with this, OPA1 functionally requires Mfn1 to regulate mitochondrial fusion but not Mfn2 [122]. Mfn1 is more efficient in mediating GTP-dependent tethering of mitochondria than Mfn2. Mfn1 also has higher GTPase activity than Mfn2, but the latter has a higher affinity for GTP [123].

Although the Mfn1 and Mfn2 genes are broadly expressed, the two genes show different levels of mRNA expression in different human tissues. Both Mfn1 and Mfn2 mRNAs are abundant in heart, and Mfn2 is also higher expressed in skeletal muscle than in other tissues [124]. In rat tissues Mfn1 protein was reported higher in heart, liver, adrenal gland and testis, whereas Mfn2 protein was expressed predominantly in the brain, but also with high levels in liver and adrenal gland [95].

#### OPA1 (ortholog of yeast Mgm1p)

OPA1, the mammalian homologue of yeast Mgm1p, is a dynamin-related GTPase that was first discovered from a gene mutation causing autosomal dominant optic atrophy [125]. The mitochondrial inner membrane-associated protein OPA1 is critical for fusion of the inner membrane and depletion of OPA1 results in small, fragmented mitochondria [122, 126]. In mammals a single OPA1 gene has at least eight transcriptional variants produced by alternative splicing [127]. The corresponding OPA1 isoforms undergo further proteolytic cleavage within mitochondria, resulting in multiple long and short OPA1 isoforms from each mRNA variant. Long OPA1 forms are integrated in the inner membrane by the N-terminal segment and short forms are associated with the inner membrane in the intermembrane space. OPA1 is essential for early embryonic survival, and homozygous inactivation of *OPA1* in mice is embryonic lethal at an early stage. Mutant fibroblasts taken from heterozygous *OPA1* mutant mice show an increase in mitochondrial fission and fragmentation [14].

#### MIEF1 promotes mitochondrial fusion in a manner that does not require Mfn2

**Fig. 6:**
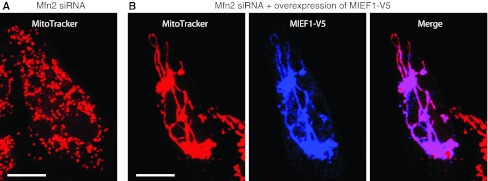
MIEF1 actively promotes mitochondrial fusion in a manner independent of Mfn2. **a** Depletion of Mfn2 by siRNA in 293T cells causes mitochondrial fragmentation. **b** Over-expression of MIEF1 in 293T cells depleted of Mfn2 reverses Mfn2 knock-down-induced mitochondrial fragmentation resulting in mitochondrial elongation. *Bars* represent 10 μm

In addition to inhibiting Drp1’s function, MIEF1 also actively promotes mitochondrial fusion in a manner that does not require the fusion-promoting factor Mfn2. It is known that depletion of Mfn2 leads to extensive mitochondrial fragmentation, whereas over-expression of MIEF1 can reverse the Mfn2 deficiency-induced mitochondrial fission, resulting in mitochondrial elongation (Fig. 6). When over-expressed in an in vivo cell fusion assay MIEF1 increases mitochondrial fusion activity [68]. Furthermore, MIEF1 distributes as punctate structures along mitochondrial tubules, which are often seen at the connection sites between two adjacent mitochondrial units and at the tips of mitochondrial tubules [68]. These observations raise the important questions whether the punctate MIEF1-containing structures on mitochondria represent fusion sites or/and inactive fission sites and how MIEF1 exerts its positive effect on mitochondrial fusion.

### Other players and regulators of the mammalian mitochondrial fusion machinery

#### OPA1 processing

Various proteases in mitochondria have been identified as involved in OPA1 processing in mammals (Table 2), including the rhomboid-related protease presenilin-associated rhomboid-like (PARL) [128], the inner membrane metalloendopeptidase OMA1/MPRP1 [129, 130], and both *m*-AAA (matrix AAA) and *i*-AAA (intermembrane space AAA) proteases in the matrix and the intermembrane space, such as the m-AAA protease subunits paraplegin [131], AFG3L1 and AFG3L2 [129], and the i-AAA protease Yme1L [132, 133]. Similar to yeast Mgm1p, OPA1 processing is required for mitochondrial fusion activity, but the mechanisms differ. OPA1 processing is little affected by the knock-out of PARL [128], whereas the yeast homologue Pcp1p, is a key protein involved in processing of Mgm1p. Although the potential homologues of mammalian Yme1L and OMA1 (Yme1p and Oma1p) are present in yeast [134, 135], their roles in Mgm1p processing are largely unknown. However, it is reported that human Yme1L can complement a yeast *yme1∆* mutant, suggesting that Yme1L is a functional homologue of Yme1p [135]. Interestingly, homologues of the mammalian m-AAA proteases, paraplegin, AFG3L1 and AFG3L2 have not been identified in yeast. Additionally, the prohibitin 1/2 (PHB1/2)-containing complexes that are localized in the mitochondrial inner membrane have been proposed to serve as scaffolds and define the spatial organization of components controlling the stability and processing of OPA1 and coordinate membrane fusion in mammals [136]. PHB1/2 are evolutionarily conserved between yeast and mammals [137, 138].

In addition to the key players Mfn1, Mfn2 and Opa1, a number of proteins have been implicated as regulators of mitochondrial fusion in mammalian cells (Table 2), including several Mfn-binding proteins such as MIB (mitofusin binding protein) [139], Stoml2 (stomatin-like protein 2, also known as SLP2) [140, 141], and the Bcl-2 family members Bax and Bak [10]. Additionally, mitoPLD (mitochondria-associated phospholipase D) [142], MICS1 (also known as GHITM, growth hormone-inducible transmembrane protein) [143] and LETM1 (leucine zipper/EF hand-containing transmembrane protein 1) [144, 145] are also involved in mitochondrial fusion. However, the mechanisms by which these proteins affect mitochondrial fusion are poorly understood.

## The PINK1/Parkin pathway is involved in mitochondrial fission/fusion dynamics

A growing number of studies indicate that *PINK1* (PTEN-induced kinase 1) and *Parkin*, the two key genes associated with autosomal recessive Parkinson’s disease (PD), are involved in the regulation of mitochondrial dynamics. PINK1 is a serine/threonine kinase and contains an N-terminal mitochondrial targeting sequence, a transmembrane domain and a C-terminal kinase domain. The protein is localized to mitochondria and spans the mitochondrial outer membrane with the N-terminal end in the intermembrane space and the C-terminal kinase domain facing the cytosol [146]. Parkin is a cytosolic E3 ubiquitin ligase with an N-terminal ubiquitin-like (UBL) domain followed by four cysteine (Cys)-rich, Zn^2+^ binding structures: really-interesting-new-gene (RING) domains RING0, RING1, in-between-RING (IBR) domain and the N-terminal RING2 [147]. Parkin is translocated to damaged mitochondria via PINK1 [148, 149]. Genetic studies in *Drosophila* indicate that PINK1 acts upstream of Parkin in a common pathway that influences mitochondrial integrity and function [150, 151]. Recent evidence suggests that the PINK1/Parkin pathway plays a crucial role in mitochondrial quality control via the autophagy machinery [147, 152].

In *Drosophila*, the PINK1/Parkin pathway is found to promote mitochondrial fission and/or inhibit fusion through interaction with the fission/fusion machinery [153, 154]. Knock-down of PINK1 or Parkin causes mitochondrial elongation, whereas over-expression of PINK1 or Parkin leads to mitochondrial fragmentation in *Drosophila* cells [155, 156]. However, several studies have reported inconsistent phenotypes in mammalian cells as compared to in *Drosophila*. Loss-of-function of either PINK1 or Parkin by siRNA treatment of cultured human cells (such as HeLa and SH-SY5Y cell lines) as well as PINK1 mutations in primary mouse neurons, and in primary fibroblasts derived from patients with autosomal recessive Parkinson disease leads to mitochondrial fragmentation. Parkin can rescue PINK1 deficiency-induced mitochondrial fragmentation, whereas over-expression of PINK1 or Parkin causes mitochondrial elongation [89, 157–160]. It has been suggested that this discrepancy is likely attributed to the different cell types used in the studies and the time point of phenotype analysis after silencing PINK1 or Parkin. For example, in primary rat post-mitotic neurons and in *Parkin* mutant fibroblasts from human PD patients, over-expression of either PINK1 or Parkin increases mitochondrial fragmentation, while knock-down of either protein causes mitochondrial elongation [161, 162]. Moreover, an increased mitochondrial fragmentation was observed as an early phenotype in *Drosophila* S2 cells depleted of PINK1 or Parkin, but this phenotype was not obvious when the cells were analyzed a longer time after siRNA treatment [157].

Further connections between the PINK1/Parkin pathway and mitochondrial dynamics are emerging. Several components in the mitochondrial fission/fusion machinery have been identified as novel substrates of the ubiquitin ligase Parkin. This sheds light on how the PINK1/Parkin pathway is involved in the regulation of mitochondrial dynamics. Parkin has been reported to regulate mitochondrial dynamics through interacting with and promoting ubiquitination and degradation of Mfn1 and Mfn2 upon induction of mitophagy [111, 163, 164]. Similarly, Parkin also promotes ubiquitination of the *Drosophila* Mitofusin (dMfn, also known as Marf in fly) [156, 165]. Notably, Drp1 was also identified recently as a substrate of Parkin in cultured human cells, in which Parkin interacts with and ubiquitinates Drp1, thereby promoting the degradation of Drp1 by the proteasome-dependent pathway. Over-expression of Parkin significantly reduces the level of Drp1, whereas knock-down of Parkin by siRNA increases the level of Drp1. However, the expression of Mfn1/2 and hFis1 was not affected by modifying Parkin levels [89]. The second ring finger domain located at the C-terminus of Parkin is required for its interaction with Drp1 and mutations derived from PD patients reduced its ability to ubiquitinate Drp1 for degradation [89]. Also, there is evidence provided by another study that hFis1 is ubiquitinated by Parkin and that over-expression of Parkin reduces the protein level of hFis1 [159]. Collectively, although there were some divergent results reported from different groups, these observations suggest that the PINK1/Parkin pathway regulates mitochondrial dynamics by the ubiquitination and degradation of either core fission or fusion proteins to selectively eliminate damaged mitochondria via mitophagy.

## Divergences in the regulation of mitochondrial dynamics between yeast and vertebrates

As several core components including Dnm1p/Drp1 and Fis1p/hFis1 of the fission machinery and Fzo1p/Mfns and Mgm1p/OPA1 of the fusion machinery are evolutionarily conserved from yeast to vertebrates and mammals, the fundamental fission/fusion machineries in mitochondrial dynamics are likely to be similar from the unicellular organism yeast to the highly complex multicellular vertebrates. In spite of this, growing evidence indicates that the co-factors and regulatory proteins, such as the Dnm1p/Drp1 interacting proteins, Mdv1p and Caf4p in yeast versus Mff and MIEF1 in human, are quite evolutionarily and functionally diverged. The yeast Mdv1p and Caf4p proteins have not been evolutionarily conserved in vertebrates, whereas Mff and MIEF1 do not exist in yeast (see also Tables 1 and 2). Given the importance of mitochondrial dynamics for many cellular processes impacting on cellular life and death and mammalian embryonic development, it is not surprising that the regulation of mitochondrial dynamics has evolved and become more elaborate in vertebrates than in yeast. This can be seen as an adaptation to meet the needs of different cell types in various tissues, particularly the needs of highly specialized cell types, such as neurons, muscle cells and sperm cells in vertebrates. For example, neurons are large polarized cells with a cell body, one long axon and multiple dendrites that arise from the cell body. Neuronal signals are transmitted along axons away from the cell body and towards synapses at the axonal terminal, and ultimately transferred to a dendrite of another neuron. Neuronal survival and functions are tightly coupled to mitochondrial dynamics [29]. It is therefore conceivable that the diversity of cell types in multicellular vertebrates requires a more elaborate set of fission/fusion machineries. It is likely that modifications of the basic theme in mitochondrial dynamics are necessary to meet the requirements of various cells types including specialized cell types in higher organisms.

**Fig. 7:**
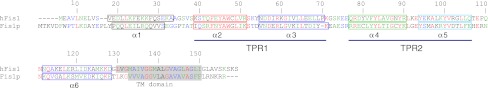
The amino acid sequence alignment of human Fis1 with its yeast ortholog Fis1p. The alignment was generated by using CLUSTALW (http://npsa-pbil.ibcp.fr/). The transmembrane domain (TM) is indicated in *gray color*. Six α-helices are indicated by *boxes*. *TPR* Tetratricopeptide repeat. The extent of amino acid similarity between hFis1 and Fis1p is indicated by *red* (identity, 22.64 % of total sequence), *green* (strongly similar, 30.82 %), *blue* (weakly similar, 12.58 %) and *black* (different, 33.96 %)

It is becoming increasingly clear that there are some differences in the control and regulation of mitochondrial dynamics in yeast and mammals. Firstly, many more proteins are involved in regulating mitochondrial dynamics in mammals than in yeast as indicated in Tables 1 and 2. Secondly, many of the regulators in mammals do not exist in yeast, while some of the regulators in yeast are not evolutionarily conserved in mammals. As an example, although OPA1 interacts with both Mfn1 and Mfn2 [166], similarly to yeast Mgm1p and Fzo1p [102, 110], the yeast linker protein Ugo1p between the inner membrane protein Mgm1p and the outer membrane protein Fzo1p has not been found in mammals. Therefore, it remains to be elucidated how Mfns and OPA1 interact during mitochondrial fusion in mammalian cells. Thirdly, although several core components seem to be evolutionarily conserved including Dnm1p/Drp1, Fis1p/hFis1, Fzo1p/Mfns, and Mgm1p/OPA1, they have in fact become quite diverged in their amino acid sequences as shown in Table 3. A comparison of the human proteins with those in other mammals, such as in mouse, show that the proteins are highly homologous at the amino acid sequence level from at least 90 % identity for Mfn1 up to 99 % for Drp1, implying that the conserved amino acids and protein domains may be required for their proper function. However, quite extensive divergences can be seen in these proteins when the comparison is made between human and yeast. As an example, yeast Fis1p and human Fis1 are only 22.6 % identical at the amino acid level, but human Fis1 is highly homologous to its orthologs in other mammals, for instance 96 % identical to mouse Fis1. Although hFis1 and Fis1p are structurally similar (Fig. 7), in that both contain a C-terminal single transmembrane domain to anchor them in the outer membrane with the bulk of the protein exposed to the cytoplasm [41, 54], and also contain cytosolic domains with six α-helices, in which the four core helices form two tandem tetratricopeptide repeat (TPR) motifs [58, 167, 168], Fis1p and hFis1 have become quite evolutionarily and functionally diverged. Structural studies indicate that the N-terminal region from Met1 to Val30 of hFis1 is the least similar to that of yeast Fis1p and the three-dimensional structures of the two proteins differ in their N-termini [47, 58, 167]. Interestingly, the N-terminal tail of Fis1p that is absent in mammalian Fis1 is required for the recruitment of Mdv1p to mitochondria [47, 167], and the Fis1p–Mdv1p interaction is a prerequisite for Dnm1p recruitment and assembly during mitochondrial fission [47]. In agreement with this, human Fis1 is unable to rescue the mutant phenotype in *fis1*∆ yeast cells [55], suggesting that hFis1 and Fis1p are not functionally interchangeable in vivo even though they are homologues in evolution. It will be interesting to learn whether Dnm1p–Drp1, Fzo1p–Mfns, and Mgm1p–OPA1 are functionally interchangeable in yeast and mammalian cells. Such comparisons may provide important insights into the convergences and divergences that have emerged during evolution of the mitochondrial dynamics processes. Fourthly, Dnm1p/Drp1 and Mgm1p/OPA1 are two key players of the mitochondrial fission/fusion machineries in both yeast and mammals. Importantly, more proteins have been found to be involved in the post-translational modifications of Drp1 and in OPA1 processing in mammals. Finally, multiple alternatively spliced variants can be produced from a single gene that controls mitochondrial dynamics in mammals, as exemplified by the eight OPA1 isoforms [127], nine Mff isoforms [65] and multiple Drp1 transcript variants [169, 170] in human. No doubt, these variants further increase the complexity by which mitochondrial dynamics potentially can be controlled and regulated in mammals, although the specific roles of the different variants in these processes require further investigation.

**Table 3:** Sequence homology comparison of the conserved mitochondrial fission/fusion proteins

Human	Mouse (identity)	Yeast (identity)
hFis1	96.1 % (Fis1)	22.6 % (Fis1p)
Drp1	98.9 % (Drp1)	43.8 % (Dnm1p)
Mfn1	90.4 % (Mfn1)	12.6 % (Fzo1p)
Mfn2	95 % (Mfn2)	12.6 % (Fzo1p)
OPA1	96.4 % (Opa1)	15.9 % (Mgm1p)

% Represents the degree of amino acid sequence identity in mouse and yeast, respectively, compared to human

## Mitochondrial dynamics and human diseases

Deregulation of mitochondrial dynamics has been associated with a wide range of pathological conditions [12], including aging [8], neurodegenerative diseases, such as Huntington’s disease (HD), Alzheimer’s disease (AD), and Parkinson’s disease (PD) [6, 20, 171, 172], diabetes [21], cardiovascular disease [173, 174], skeletal muscle atrophy [24], and cancer [26, 175].

### Mitochondrial dynamics in neurodegeneration

Neurons are particularly sensitive to changes in mitochondrial function since they have limited glycolytic capacity, which makes them particularly mitochondria-dependent for their supply of energy. In addition, they are extremely metabolically active, i.e., synaptic transmission, axonal/dendritic transport, ion channels and ion pump activities are all energy taxing processes [176]. Not surprisingly, rapidly growing evidence indicates that deregulation of mitochondrial dynamics results in neuronal dysfunction and thus contributes to neuronal injury and death in many neurodegenerative diseases [4, 6, 12]. Mutations in genes encoding components of the mitochondrial fission/fusion machineries have been linked to human neurodegenerative diseases: Mfn2 in Charcot-Marie-Tooth neuropathy type 2A (CMT2A) [177], OPA1 in autosomal dominant optic atrophy (ADOA) [125, 178], GDAP1 in Charcot-Marie-Tooth neuropathy type 4A (CMT4A) [179, 180] and Drp1 in abnormal brain development, optic atrophy and neonatal lethality [17]. Alzheimer’s disease-associated amyloid-β (Aβ) derived from amyloid precursor protein (APP) triggers *S*-nitrosylation of Drp1 (forming SNO-Drp1) resulting in mitochondrial fragmentation and dysfunction, contributing to the Aβ-mediated pathogenesis of AD [90]. Likewise, Parkinson’s disease-associated gene products (Parkin and PINK1) can not only influence the morphology of mitochondria, but also regulate mitochondrial degradation by mitophagy and possibly the transport of mitochondria in axons. Huntington’s disease is an autosomal dominant disease caused by mutations that result in trinucleotide expansion (CAG) within a single gene, Huntingtin (Htt). Mutant Htt can induce mitochondrial fission, and expression of either dominant-negative Drp1 or Mfn2 prevents this change in mitochondrial phenotype [181]. Collectively, disruption of mitochondrial dynamics may represent a common pathway leading to neuronal dysfunction, and thus the mitochondrial fusion/fission machinery may provide new therapeutic targets in patients with neurodegenerative diseases.

### Mitochondrial dynamics in cancer

Cancer cells manifest two particular mitochondrial properties distinct from healthy cells [182]. First, mitochondria in cancer cells exhibit relative resistance to the induction of mitochondrial membrane permeabilization, which is one of the decisive steps of the intrinsic apoptotic pathway [183]. Evading apoptosis constitutes one of the essential hallmarks of cancer [184]. Therefore, one obstacle of cancer therapy is the development of cancer resistance to chemotherapy. The molecular mechanisms by which the resistance is developed remain largely unknown. Second, mitochondria have been implicated in regulation of the cellular energy metabolism in cancer cells [185]. In normal cells, ATP is mainly supplied by mitochondrial oxidative phosphorylation. However, in cancer cells, mitochondria exhibit a reduced oxidative phosphorylation, thus the cellular energy metabolism shifts towards ATP generation through glycolysis (Warburg effect). Increased aerobic glycolysis has been observed in various tumor cells.

Mitochondria undergo extensive fragmentation during apoptosis. Although this fragmentation appears to be universally associated with apoptosis, extensive mitochondrial fission can occur in the absence of apoptosis. However, many studies support that the mitochondria-shaping components of the fission/fusion machinery are directly involved in the regulation of cell death pathways including apoptosis, cellular aging and autophagy [10]. Mitochondrial fusion usually confers resistance to apoptosis, whereas mitochondrial fission makes cells more sensitive to apoptotic stimuli [10, 186]. A number of reports have shown that down-regulation of either Drp1 or hFis1 leads to mitochondrial elongation and leads to cellular resistance to various apoptotic stimuli, whereas silencing of Mfn1, Mfn2 or OPA1 results in mitochondrial fragmentation and an increased sensitivity to apoptotic stimuli [10, 187]. However, it is unclear whether cancer cells can modify mitochondrial dynamics to acquire resistance to apoptosis [26]. One study reveals that the apoptosis repressor with caspase recruitment domain (ARC) contributes to cancer cell’s resistance to chemotherapy by inhibition of mitochondrial fission mediated by the fission protein Drp1 [188]. We recently reported that MTGM, a regulator of mitochondrial dynamics, is up-regulated in human brain tumors [74]. A more recent study reported that Drp1 is up-regulated and Mfn2 down-regulated in both human lung cancer cells and lung tumor tissues from patients. As a result, lung cancer cells contain more fragmented mitochondria than healthy lung cells. Notably, increasing expression of Mfn2 or inhibiting Drp1 can reduce tumor growth and increase spontaneous apoptosis in a xenotransplantation model [189]. These data imply the potential importance of dysregulated mitochondrial dynamics in the pathogenesis of human cancer and its potential role as a therapeutic target in human cancer. Apparently, as a starting point for understanding the importance of mitochondrial dynamics in cancer, further studies are required to elucidate whether an imbalance between mitochondrial fission and fusion is present in different types of human cancer and how mitochondrial dynamics and mitochondria-shaping proteins are involved in the energy metabolism, growth, and resistance of cancer cells to chemotherapy. Several studies have shown that mutations and aberrant expression of mitochondria-shaping proteins are closely associated with the reduction of mitochondrial respiration and ATP generation [6, 7, 9, 119]. Mitochondrial dynamics is likely involved in the shift of cellular energy metabolism towards ATP generation through glycolysis in cancer cells. Therefore, mitochondrial dynamics may represent an important field of developing new molecular targets for drug development and therapeutic intervention of human cancer and other diseases. No doubt, novel insights into the complex crosstalk between mitochondrial dynamics and cellular physiology are likely to not only have great potential for future scientifically fascinating discoveries but, from a more practical perspective, also in clinical medicine.

## In summary

Although the key players (Drp1, hFis1, Mff, MIEF1, Mfn1/2 and OPA1) in the mitochondrial fission/fusion machineries and a number of regulatory proteins have been identified in mammals, the exact molecular mechanisms controlling mitochondrial fission and fusion are still largely unknown, and more work is required to identify additional proteins involved in mitochondrial dynamics. An important challenge for future studies is to determine the potential interactions between the key players and their regulators, and to place these players with their regulators into (a) comprehensive pathway(s) to learn how the cell can regulate these processes, as well as to learn how the mitochondria-shaping proteins impact on various cellular biological processes and human diseases. It is clear that we are beginning to recognize the extensive divergences in the regulation of mitochondrial dynamics between the unicellular organism yeast and multicellular vertebrates. These differences may provide further options to experimentally steer the process in mammals, and this may be of major importance for example in research on cancer, diabetes, cardiovascular, and neurodegenerative diseases.
